# Preliminary cross-sectional reliability and validity of the Skull Base Inventory (SBI) quality of life questionnaire

**DOI:** 10.1186/s40463-016-0158-y

**Published:** 2016-09-07

**Authors:** Soroush Larjani, Eric Monteiro, Ian Witterick, Allan Vescan, Gelareh Zadeh, Fred Gentili, David P. Goldstein, John R. de Almeida

**Affiliations:** 1 Department of Otolaryngology Head and Neck Surgery, University Health Network, Toronto, ON Canada; 2 Department of Otolaryngology Head and Neck Surgery, Mount Sinai Hospital, Toronto, ON Canada; 3 Department of Neurosurgery, University Health Network, Toronto, ON Canada; 4 Princess Margaret Cancer Center, 610 University Avenue, 3-955, Toronto, M5G 2 M9 ON Canada

**Keywords:** Quality of life, Questionnaire, Skull base surgery, Endoscopic, Open

## Abstract

**Background:**

The Skull Base Inventory (SBI) was developed to assess the quality of life of patients undergoing endoscopic or open approaches for anterior and central skull base pathologies. In this study, we sought to establish the discriminative and evaluative properties for this instrument.

**Methods:**

The SBI was administered in a cross-sectional fashion to patients who previously had skull base surgery after treatment and then again 2 weeks after completing the instrument. Internal consistency, test-retest reliability, and construct validity were determined. Four constructs were evaluated with the following a priori hypotheses: lower scores will be seen in patients with 1.malignant versus benign histology, 2.a history of radiation versus none, and those with 3.recurrences versus no recurrence, and 4.items deemed relevant versus irrelevant by respondents.

**Results:**

Fifty-two patients completed the questionnaire; 32 had endoscopic and 20 open surgeries. Internal consistency was good (>0.7 and <0.95) for all domains except one. Test-retest reliability was good (>0.70) for 38 of 41 items. Four constructs were evaluated and three were consistent with a priori hypotheses (*p* < 0.05). The instrument failed to confirm the hypothesis that malignant tumours are associated with poorer scores than benign.

**Conclusions:**

The SBI demonstrated preliminary reliability and validity for discriminative use.

## Background

Skull base tumours pose a significant challenge in surgical oncology due to their proximity to vital anatomical structures [1]. In many instances, the primary management of these tumours is surgical resection. Given the challenging anatomical locations of such tumours, patients may experience significant morbidity secondary to the disease itself and/or the treatment [2–4]. With recent improvements in endoscopic and open surgical approaches, there has been an increasing interest in the impact of tumours and surgical resection on patients’ health-related quality of life (QOL) [5]. QOL studies can help inform health care providers of the functional limitations that patients face, the obstacles to returning back to their pre-illness health state, and the magnitude of quality of life impairments by treatment approach.

To date, a number of QOL instruments for head and neck cancers have been developed [2, 6–8]. Very few have been designed to measure the QOL of patients with anterior and central skull base pathologies. The existing instruments are also limited in capturing the unique morbidities that result from different tumour pathologies, locations, and surgical approaches. The Skull Base Inventory (SBI) was designed to overcome the limitations of existing instruments [9].

The SBI is a multidimensional, disease-specific instrument designed to measure QOL of patients who undergo surgical treatment, regardless of surgical approach, for anterior or central skull base pathologies [9]. The instrument has been designed using both expert and patient input to be applicable to those who undergo open or endoscopic surgical approaches. The SBI was designed for both discriminative and evaluative purposes, to capture the temporal change in QOL, and to differentiate between the QOL of different patient populations, which is of particular importance for assessing various surgical modalities. It consists of 41 questions covering 11 disease-specific domains including social, emotional, physical, cognitive, family, financial, spiritual, endocrine, nasal, neurologic, and visual. The comprehensive measurement properties of the SBI however, have not yet been evaluated. The purpose of the present study is to evaluate the psychometric properties of the SBI in a cross-sectional study. In specific, we sought to determine the instrument’s reliability and validity in capturing disease-specific domain and overall QOL of patients who have undergone skull base surgery.

## Methods

### Patient population

This study was granted approval by the University Health Network’s Research Ethics Board. Informed consent was obtained from all included patients.

All patients who were surgically treated for anterior or central skull base pathologies between 1998 and 2008 at the University Health Network were screened as potential participants for this study. Patients were included if they had a minimum of 6 months from time of surgery, were English speaking, currently alive, not lost to follow-up, and were willing to consent to study participation. Patients were contacted by telephone, interviewed over the phone, and asked to complete the questionnaire by interview. This study was executed in a cross-sectional fashion with patients recruited between January and June 2009.

### Instrument administration and chart review

Included patients were asked to complete the 41 item questionnaire by telephone and were subsequently asked to complete the questionnaire again 14 days after their initial completion in order to assess test-retest reliability. Although there is no evidence for the optimum time interval between test administration in test retest reliability of health status instruments, we chose 14 days to be long enough to minimize subject recall and carryover effect, while having the time period short enough to reduce the chance for change in health and/or mood status [10]. In addition, patients were asked to rate the relevance of each item on a seven point Likert scale to their overall quality of life. The instrument scoring has previously been described [9]. In brief, each domain is scored out of 100 with lower scores associated with poorer quality of life. Items within each domain for which a response was recorded were summed and averaged out of 100. A composite score assuming equivalent weighting of domains in overall quality of life was created by averaging domain scores. A retrospective chart review was conducted for patients who consented to participate in this study, and the following data were collected: patient factors (age, gender), disease factors (histopathology, tumour location, stage) and type of adjuvant/neoadjuvant therapies (radiotherapy, chemotherapy).

### Statistical analysis

The consensus-based standards for the selection of health measurement instruments (COSMIN) guidelines for assessing the quality of studies on measurement properties of health measurement instruments was reviewed, and the following statistical analyses were performed to establish the discriminative and evaluative properties of the SBI [11]. Internal consistency within domains was assessed using Cronbach’s alpha. Internal consistency was rated from unacceptable to acceptable as previously described [12]. Item test-retest reliability was performed using intra-class correlation (ICC). Correlation coefficients of 0.7 or higher were considered good test-retest reliability. Validity testing was performed using construct validity. Four constructs with the following a priori *hypotheses* were tested: (1) patients with malignant pathologies have lower scores than those with benign pathologies (2) patients with a history of radiation have lower scores than those with no history of radiation, (3) patients with tumour recurrence will have lower scores than those with no recurrence, and (4) items deemed relevant by respondents will be associated with lower scores than those deemed irrelevant. All 41 questions were examined for floor and ceiling effects; floor and ceiling effects were considered when more than 20 % of participants chose the lowest or highest possible score, respectively. The minimally important clinical difference (MCID) was determined using distribution method (MCID = 0.5 standard deviation) [13]. Probability (*p*) values ≤ 0.05 were considered statistically significant. All statistical analyses were performed using IBM SPSS 20.0.

## Results

### Patient demographics

Fifty-two patients completed the 41-question SBI. Patient, disease, and treatment factors are described in Table 1. The group of tumours consisted of both anterior and central skull base locations. Tumour histopathologies varied considerably, and included adenocarcinoma (5.8 %), adenoid cystic carcinoma (1.9 %), anterior skull base/olfactory groove meningioma (3.8 %), planum or tuberculum meningioma (5.8 %), cavernous hemangioma (3.8 %), hemangioma (1.9 %), hemangiopericytoma (1.9 %), chondrosarcoma (1.9 %), chordoma (17.3 %), craniopharyngioma (7.7 %), esthesioneuroblastoma (13.5 %), corticotropic pituitary adenoma (13.5 %), somatotropic pituitary adenoma (7.7 %), gonatotropic pituitary adenoma (1.9 %), lactotropic pituitary adenoma (3.8 %), juvenile nasopharyngeal angiofibroma (1.9 %), leiomyosarcoma (1.9 %), osteosarcoma (1.9 %), and squamous cell carcinoma (1.9 %).

**Table 1:** Demographics

Patient Factors	
Average age (years)	46.4 ± 14.7
Gender	
Male	24 (46.1 %)
Female	28 (53.8 %)
Disease Factors	
Disease Status	
Primary	41 (78.8 %)
Recurrent	11 (21.2 %)
Tumour Location	
Anterior	22 (42.3 %)
Central	30 (57.7 %)
Benign vs. Malignant	
Benign	27 (51.9 %)
Malignant	25 (48.1 %)
Treatment Factors	
Surgical approach	
Endoscopic	32 (61.5 %)
Open	20 (38.5 %)
Radiotherapy	
Pre-op	17 (32.7 %)
Post-op	6 (11.5 %)
Chemotherapy	
Pre-op	1 (1.9 %)
Post-op	2 (3.8 %)

### Reliability

Internal consistency of all domains of the SBI are presented in Table 2. The ‘other’ domain contains items generally felt to be unrelated and comprised a type of miscellaneous domain thus explaining the unacceptable rating for internal consistency. The spiritual domain also had an unacceptable rating for internal consistency.

**Table 2:** Internal consistency

Domain	α (95 % CI)	Rating
Cognitive	0.75 (0.60–0.85)	Acceptable
Emotional	0.94 (0.90–0.96)	Excellent
Family	0.85 (0.75–0.91)	Good
Financial	0.80 (0.65–0.89)	Good
Social	0.77 (0.62–0.86)	Acceptable
Spiritual	0.33 (−0.23–0.63)	Unacceptable
Endocrine	0.60 (0.38–0.75)	Questionable
Nasal	0.71 (0.55–0.82)	Acceptable
Neurologic	0.66 (0.49–0.79)	Questionable
Visual	0.75 (0.62–0.85)	Acceptable
Other	0.20 (−0.19–0.50)	Unacceptable
All domains	0.87 (0.80–0.92)	Good

Test-retest reliability for 38 out of 41 questions was good (≥0.7). Three items had a test-retest reliability less than 0.7, two of which were in the visual subdomain (ICC = 0.66, 0.66) and one of which was in the social domain (ICC = 0.42).

### Construct validity

**Fig. 1:**
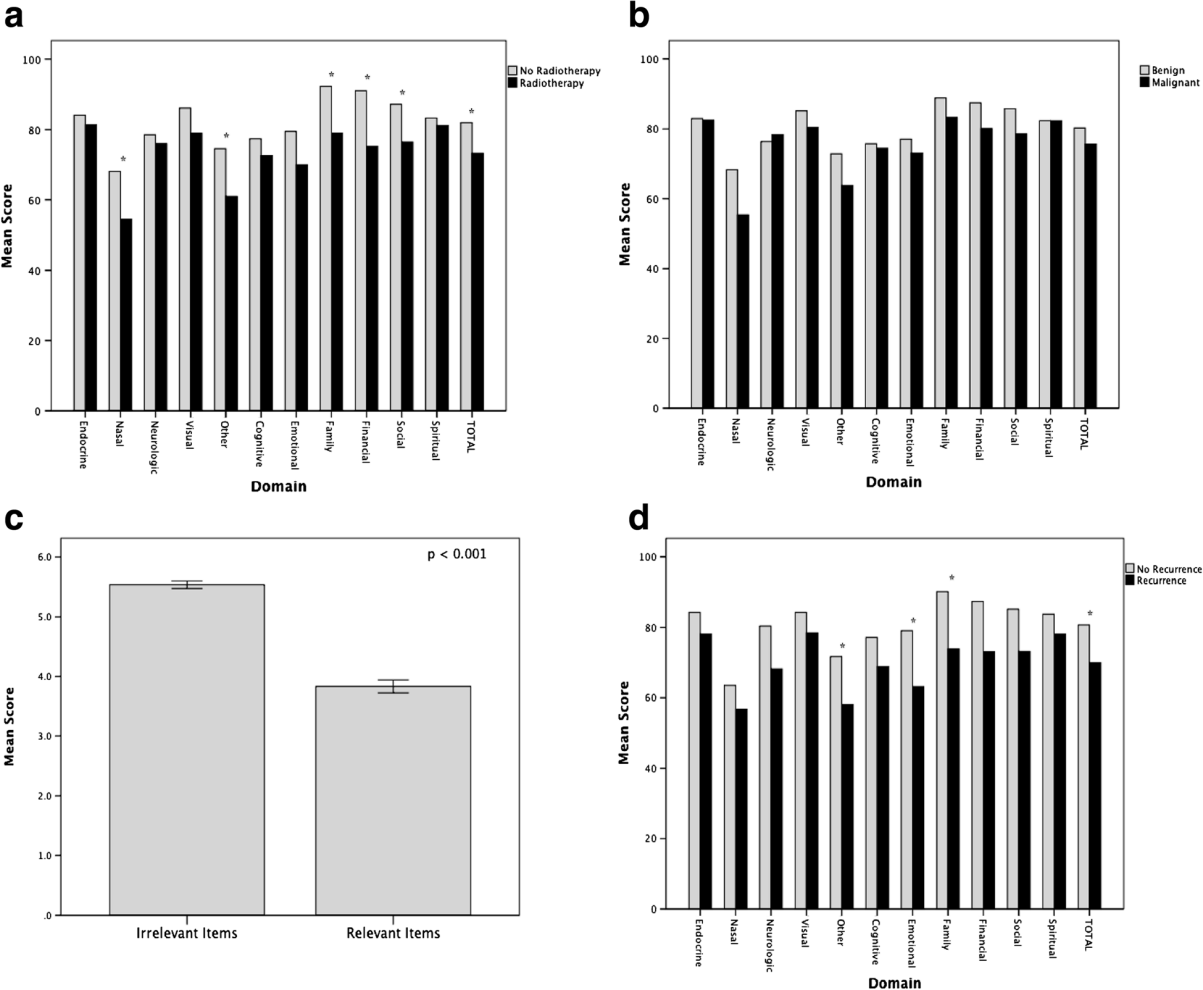
Average SBI scores of different QOL domains for patients with and without radiotherapy (**a**), malignancy (**b**), and recurrent disease (**d**). Average scores for items on the SBI deemed relevant versus irrelevant (**c**)

Using the SBI, four constructs were evaluated (Table 3). Three of the four constructs tested matched the a priori hypotheses. Patients with a history of radiotherapy had a worse overall QOL than those who did not receive radiotherapy (73.3 vs 82.0, *p* = 0.04) with statistically poorer QOL in family (79.0 vs 92.3, *p* = 0.03), financial (75.3 vs 91.1, *p* = 0.03) social (76.5 vs 87.2, *p* = 0.05), and nasal domains (54.5 vs 68.1, *p* = 0.05) (Fig. 1a). Patients who had recurrent tumours also had worse overall QOL than those who did not (70.0 vs 80.7, *p* = 0.03); with lower domains scores in the emotional (63.2 vs 79.1, *p* = 0.05), family (73.9 vs 90.2, *p* = 0.02), social (73.2 vs 85.2, *p* = 0.05), and neurologic (68.2 vs 80.4, *p* = 0.05) domains (Fig. 1b). The fourth construct examined demonstrated that items on the questionnaire that were deemed irrelevant by patients were associated with better scores than those that were deemed relevant (*p* < 0.001) (Fig. 1c). Contrary to the a priori hypothesis, patients with malignant tumours did not have a statistically significant difference in overall QOL compared to those with benign tumours (75.7 vs 80.3, *p* = 0.28) (Fig. 1d). The average time after surgery for completion of the SBI for those with benign disease was 47.7 months versus 112.7 months for those with malignant disease (*p* < 0.0001).

**Table 3:** Difference in QOL of different patient populations (*p* values)

	(1) Malignant vs benign	(2) History of radiation vs none	(3) Recurrence vs none
Cognitive	0.83	0.43	0.24
Emotional	0.56	0.17	0.05^a^
Family	0.36	0.03^a^	0.02^a^
Financial	0.32	0.03^a^	0.09
Social	0.18	0.05^a^	0.05^a^
Spiritual	1.00	0.65	0.30
Endocrine	0.92	0.57	0.29
Nasal	0.06	0.05^a^	0.40
Neurologic	0.73	0.65	0.05^a^
Visual	0.37	0.18	0.35
Other	0.09	0.01^a^	0.03^a^
All domains	0.27	0.04^a^	0.03^a^

^a^ Statistically significant

### Floor and ceiling effects

No floor effects were observed for any of the items on the SBI. Ceilings effects (>20 %) were observed in the following 6 domains: emotional, family, financial, spiritual, endocrine, and visual (Table 4). The financial and family domains had ceiling responses in 56 % and 52 % of respondents, respectively. The MCID was determined to be 0.82.

**Table 4:** Floor and ceiling effects

Domain	Floor effect (% with lowest possible score)	Ceiling effect (% with highest possible score)
Cognitive	No (0)	No (14)
Emotional	No (0)	Yes (25)
Family	No (0)	Yes (52)
Financial	No (2)	Yes (56)
Social	No (0)	No (14)
Spiritual	No (0)	Yes (29)
Endocrine	No (0)	Yes (23)
Nasal	No (0)	No (6)
Neurologic	No (0)	No (14)
Visual	No (0)	Yes (23)
Other	No (0)	No (6)

## Discussion

Measuring QOL in patients with skull base neoplasms is challenging because of the variety of changes experienced as a result of the pathology as well as the treatment. The World Health Organization defines quality of life as ‘individuals’ perception of their position in life in the context of their culture and value systems in which they live in and in relation to their goals, expectations, standards, and concerns [14]. It is a broad ranging concept affected in a complex way by the person’s physical health, psychological state, level of independence, social relationships, personal beliefs, and their relationship to salient features of their environment” [14]. In addition to affecting ones physical functioning, treatment for skull base neoplasms affects several other domains of life [9]. We designed an instrument which purports to measure changes in QOL for patients with skull base neoplasms. Herein we present the first psychometric assessment of the SBI and evaluation of the measurement properties of this instrument. The study incorporated responses from participants who had undergone skull base surgery at different time points with a wide range of histopathologies, anatomic locations (anterior and central skull base) and surgical approaches (endoscopic and open). Our results demonstrate that the SBI has sound reliability and validity in capturing QOL in disease specific domains and overall QOL in patients who undergo skull base surgery, regardless of when the instrument was completed with respect to the operation. The only domain that had an unacceptable rating for internal consistency was the spiritual domain. In the SBI, there are two questions within the spiritual domain: (1) In the past 2 weeks, has your condition made you question your religious beliefs? and (2) How would you rate your ability to appreciate the small things in life. These two questions may or may not be of the same measure and this highly depends on one’s personal belief system, thoughts, opinions, and perceptions of the world around them; therefore explaining the lack of internal consistency observed within this domain. In the present study, we did not have sufficient sample size to perform factor analysis. In future studies we will perform factor analysis to determine if items load well onto each domain to confirm the items belong in the proposed domains.

We tested construct validity through four a priori *hypotheses*. Three of these hypotheses were confirmed: (1) patients with a history of radiation have a worse QOL, (2) patients with primary cancer recurrence have a worse QOL, and (3) questions on the SBI that have little relevance to patients disease state are consistently scored high. However, results obtained rejected our hypothesis of patients with malignant tumours having worse QOL than those with tumours of benign pathology. Failure to verify this construct may be due either to the fact that there indeed is no difference in QOL between patients with malignant or benign tumours in our cohort, there is a difference but the sample size was inadequate to detect a difference, or there is a difference but the instrument was not sensitive enough to detect this difference. The timing after surgery at which the questionnaire was completed may also explain the failure to detect this difference. Previous studies have shown a difference in QOL between malignant and benign skull base tumours at 6 months after surgery [15]. However, the QOL impairment shortly after surgery, seems to improve over time. In our cohort, patients completed questionnaires generally much later after their surgery. As such it may be that with time there is a normalization of QOL that makes patients with benign and malignant pathologies have similar QOL.

In the last two decades, anterior skull base surgery has evolved rather rapidly owing to improved surgical techniques [16, 17]. Advancements have led to reduced mortality and morbidity. With the advent of endoscopic approaches, comparisons have been made with open approaches in terms of the extent of surgical resection, survival, and now, QOL [15–17]. In general, most studies focusing on QOL have found that endoscopic approaches allow for earlier improvements in QOL following surgery [18, 19]. Nevertheless, open approaches continue to be necessary for tumours that are not amenable to endoscopic resection and depending on surgeon preference.

There are a number of challenges in measuring the QOL of patients with skull base pathologies. Skull base tumours are situated within the vicinity of vital neurovascular structures that play significant roles in different sensory organs and cognition. Endoscopic and open surgical approaches also result in different post-operative morbidities. We have shown that the symptom profiles of different tumours vary considerably owing to variation in size, morphology, location, pathology, and the surgical technique used for resection [20]. Although it is ideal to have different QOL instruments for different tumour pathologies and locations, this is simply not feasible due to the vast number of possible combination of tumours types and locations. It is therefore important to have a site-specific instrument that can accurately and holistically capture sensory, physical, and cognitive symptoms following skull base surgery, and be applicable to both endoscopic and open surgical approaches for different tumour pathologies.

The Anterior Skull Base questionnaire (ASB) is an existing instrument for measuring QOL after skull base surgery. It is a validated and reliable tool that has six domains [2, 21]. The ASB has been shown to be able to predict postoperative QOL after skull base surgery [22]. However, the ASB was originally developed for open skull base surgical patients. It consists of only seven disease-specific items that may limit its discriminative ability for different populations. In contrast, the SBI consists of 26 disease specific items along with five physical subdomains and one cognitive domain that may increase its discriminative ability; this must be tested in future comparative studies.

Based on the COSMIN guidelines for assessing the methodological quality of studies on measurements properties of health status instrument, a comprehensive instrument must consist of three domains: reliability, validity, and responsiveness [10]. In this cross-sectional study, we were limited in only assessing reliability and validity since our study population comprised of patients who had already undergone surgery and were measured in a cross-sectional fashion. Additional multi-institutional prospective studies are required to assess the responsiveness of the instrument by capturing the QOL of patients before and after surgery, and evaluating the change in QOL over time. Furthermore, a number of aspects of validity were not evaluated, such as cross-cultural validity, which is critical in QOL studies as perception of varying health status’ can differ considerably for patients of different socio-cultural backgrounds. Validating such properties of the instrument require additional studies with different patient populations and at different institutions.

## Conclusion

Our preliminary data suggests that the SBI has sound reliability and validity in assessing disease-specific and overall QOL of patients who undergo skull base surgery for anterior and central pathologies with both endoscopic and open surgical approaches. Additional studies are needed to further validate the psychometric and evaluative properties of the instrument in different patient populations, and of different institutions.
